# Dietary B vitamins influence the *Drosophila melanogaster* preference for dietary yeast

**DOI:** 10.17912/micropub.biology.001354

**Published:** 2024-12-05

**Authors:** Dean B Peterson, John Chaston, Andrew T Call

**Affiliations:** 1 Department of Plant and Wildlife Sciences, Brigham Young University, Provo, UT, United States

## Abstract

The microbiota influences the *Drosophila melanogaster* dietary preference for yeast (DPY). We previously identified four transposon insertion mutants in *Acetobacter fabarum* that significantly influence fly DPY, and three of these insertions were in genes that are associated with thiamine metabolism. Here, we tested if thiamine influences fly DPY in monoassociated flies. We show that thiamine and other B vitamins influence fly DPY and that the different mutants have distinct DPY responses to thiamine supplementation. Together, these experiments identify specific nutritional effectors of *D. melanogaster* DPY.

**Figure 1. Thiamine influences D. melanogaster DPY.**
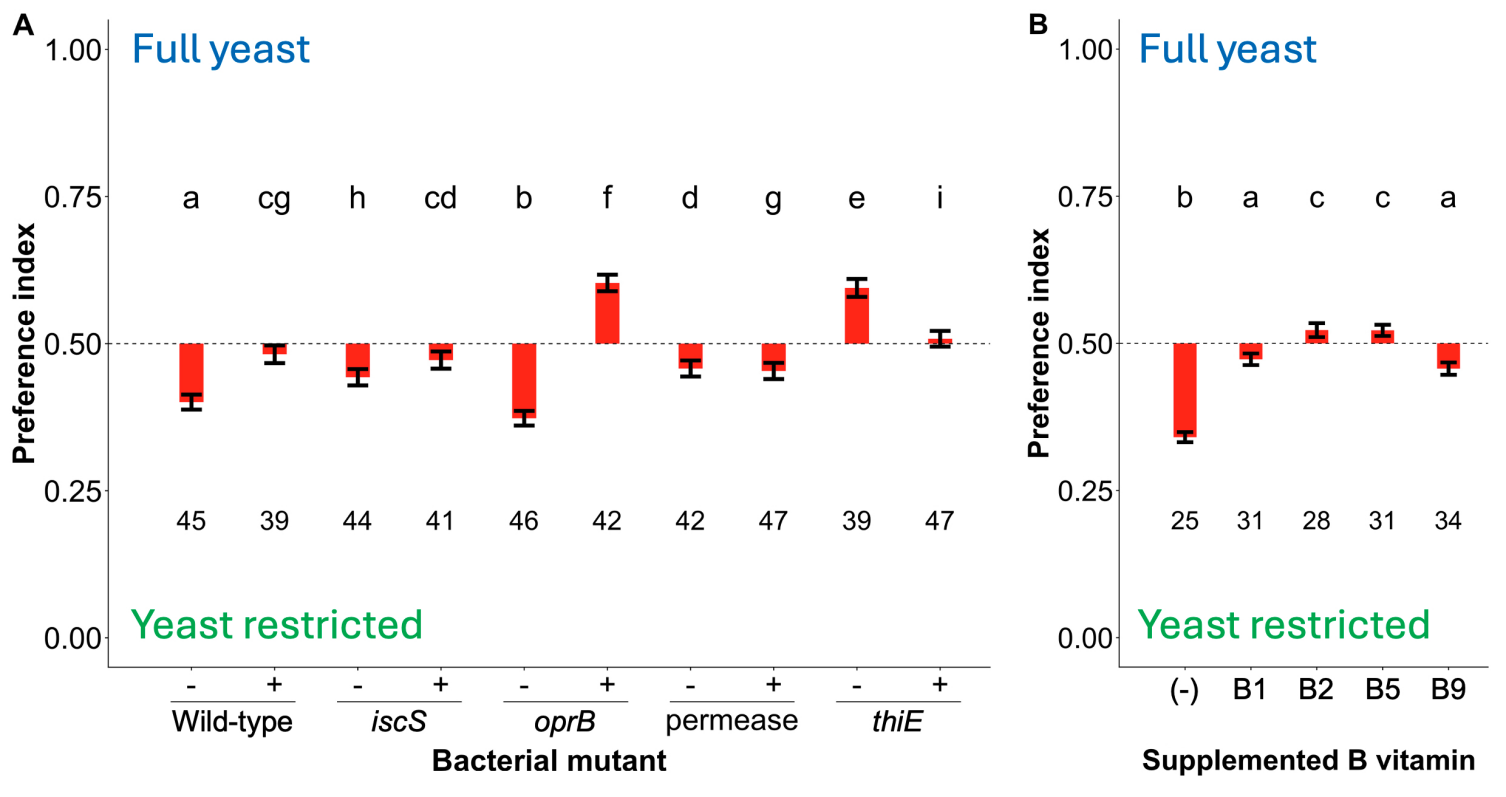
We measured the number of times individual flies fed from each of two diet troughs, one containing standard “control” diet (full-yeast) and the other containing trial diet (yeast-restricted) in a flyPAD arena for 60 minutes. A) Flies were monoassociated with bacterial mutants and reared on yeast-glucose (Y-G) diet that was (+) or was not (-) supplemented with 1.4 mg L ^-1^ thiamine. B) Flies were monoassociated with *A. fabarum* and reared on Y-G diet supplemented with different B vitamins. Different letters above the columns represent statistically significant differences between treatments as determined by a generalized linear mixed effects model with a binomial family with a post hoc Tukey test. The number of individually tested flies is reported below the bars, and at least three separate experiments in time were conducted on each condition. The error bars represent the standard error for the mean. The data are shown as the average fly preference index, which was extracted from the model as the linear prediction of the inputted values. Gene abbreviations and names are reported in Table 1.

## Description

Associated microorganisms (“microbiota”) impact their host’s life history and behaviors. This study used *Drosophila melanogaster* fruit flies as a model to better understand the impact of microbiota on their host’s dietary preference to feed on yeast (DPY). Dietary preferences can be observed if an organism consumes more of one diet than another. As a model, the *D. melanogaster* microbiota is of low taxonomic diversity and numerical abundance, and its members are easily grown in laboratory culture (Wong et al., 2011; Chandler et al., 2011; Staubach et al., 2013). In *D. melanogaster* , diet preference can be influenced by the flies’ mating status (Camus et al., 2018) , temperature (Brankatschk et al., 2018) , essential amino acid (eAA) deprivation (Leitao-Gonçalves et al., 2017; Henriques et al., 2020), chronic hypoxia (Vigne et al., 2010) , and microbiota composition (Wong et al., 2017; Henriques et al., 2020; Leitao-Gonçalves et al., 2017). In the laboratory, the microbiota reduces the flies preference to consume yeast. In flies reared on a holidic laboratory medium, a two-species community of a lactic acid bacterium (LAB) and an acetic acid bacterium (AAB) caused reduced DPY (Leitao-Gonçalves et al., 2017; Henriques et al., 2020); in flies reared on nutritionally-rich but undefined medium made up of yeast and glucose, a variety of LAB and AAB were sufficient to lower fly DPY (Call et al., 2022) . Although AAB can provide eAA's to promote lactate production in their companion LAB strains, eAA's are not the AAB molecules that suppress fly DPY (Leitao-Gonçalves et al., 2017). Instead, it is likely that AAB produces molecules – the identities of which are not currently known – from their metabolism of LAB-produced lactate to influence their hosts’ feeding preferences (Henriques et al., 2020) . Our recent work using transposon insertion mutants of the AAB strain *A. fabarum* showed that fly DPY was altered when transposons were inserted into genes associated with thiamine metabolism (Call et al., 2022) . Here, we sought to test the hypothesis that thiamine might be responsible for bacterial suppression of fly preference to consume yeast.

To test if thiamine reduces the fly DPY, we reared flies monoassociated with wild-type and mutant *A. fabarum* on a standard laboratory diet supplemented with thiamine. As controls, we confirmed that flies reared without supplemented thiamine generally showed the same trends observed previously ( Fig. 1A ) (Call et al., 2022) . Relative to the wild-type bacterial strain, flies colonized by three mutants (transposon insertions in *iscS* , *thiE,* and a permease gene) had increased DPY. Supplementing the flies’ diet with thiamine suppressed the wild-type *A. fabarum-* dependent restriction of fly DPY (Fig 1A) and the effect of two mutations (insertions in *iscS* and a permease gene), suggesting that thiamine could be a molecule that potentiates fly DPY. Counter to this expectation, thiamine supplementation reduced fly DPY in a *thiE* mutant. This discordant observation can be explained if thiamine supplementation influences fly DPY indirectly rather than as a direct effector. Regardless, our results show that the presence of additional thiamine in the diet influences fly DPY.

In addition to thiamine-related genes, our previous work also showed that insertions in *oprB* influenced fly DPY. The normal effect of a transposon insertion in *oprB* , which encodes a glucose permeable porin (Wylie et al., 1995) , is to reduce fly DPY, although the effect is not by modifying the protein to glucose ratio in the fly diet (Call et al., 2022) . When reared on diet supplemented with thiamine, the magnitude of the shift in DPY was more dramatic than in any other condition we tested. This may suggest that the functions of this transporter are related to the thiamine condition of the flies’ diets, or to a molecule whose abundance in flies, microbes, or the dietary environment is affected by dietary thiamine levels.

To test if the DPY effect on *D. melanogaster* was specific to thiamine, we reared monoassociated flies on diet inoculated with thiamine or individually with other B vitamins (thiamine/B1, riboflavin/B2, pantothenic acid/B5, folic acid/B9) (Fig 1B). We found that each of the B vitamins we tested increased fly DPY, albeit to different extents. Therefore, we conclude that there is a non-specific effect of thiamine supplementation on fly DPY; however, we did not test if the other various B vitamins conferred shared or distinct responses when paired with the different mutants.

In summary, these results reveal that diets supplemented with thiamine or other B vitamins counteracted the *A. fabarum* mediated suppression of fly DPY. It is not yet clear why we detected a relatively broad impact of B vitamins on the fly DPY, whereas a previous analysis reported that vitamins do not influence fly feeding preference for yeast (Leitao-Gonçalves et al., 2017). A possible explanation for the difference in outcome is that the previous study subtracted these nutrients from a chemically defined diet (Piper et al., 2014) , whereas we measured the influence of supplementing nutrients to a nutrient-rich, undefined yeast-glucose diet. We also used a distinct fly genotype and different bacterial strains than previous experiments. Finally, even though we used the same device to measure feeding preferences, our analysis focused on a preference index shift, rather than the consumption of low-yeast diet. When analyzing raw sip-count data there was no significant difference in the consumption from the low-yeast troughs in the different conditions in our study, reinforcing that comparing preferences to direct consumption can lead to distinct outcomes. We have not yet investigated if other B vitamins also alter the influence of the bacterial mutants on fly DPY (e.g. to suppress the effect of an *iscS* or permease gene mutation). In future experiments, flies colonized by bacterial mutants with site-directed deletions of the target genes could verify the identity of the gene or genes influenced by the transposon insertions we studied here. Another area that could be investigated is whether pH of the diet in the flyPAD influences how thiamine and other B vitamins counteract the microbiota mediated suppression of fly DPY. Fly feeding can be influenced by dietary pH (Chen et al., 2014; Deshpande et al., 2015) , but we omitted acid additives in the flyPad because acid was omitted in the initial screen we are following on. We expect that further exploration of the interactions between different nutrients, *D. melanogaster* dietary preferences, and the microbiota will lead to greater understanding of how the microbiota can influence complex animal behaviors.

## Methods

**Fly maintenance and diet preparation.** A *D. melanogaster* CantonS stock originally obtained from Mariana Wolfner was used in all experiments. Our stock is not colonized by the reproductive tract endosymbiont, *Wolbachia* . Flies were reared on a yeast-glucose (Y-G) diet (10% Brewer’s Yeast [0290331225; MP Biomedicals], 10% a-D-Glucose [158968-5KG; Sigma], 2% agar [903312; MP Biomedicals], and double distilled H _2_ O boiled together and cooled to 60°C; 0.04% phosphoric and 0.42% propionic acid added after cooling) in an incubator at 25°C on a 12-h light:12-h dark cycle at ambient atmosphere (approx. 25% humidity).

**Monoassociated *D. melanogaster* rearing conditions.** To rear monoassociated flies we inoculated bacteria-free embryos with *A. fabarum* wild-type or mutant strains as in our previous work (Koyle et al., 2016) . Briefly, we harvested *D. melanogaster* eggs from grape-juice agar plates (Y-G diet with 140mL L ^-1^ Welch’s frozen grape juice concentrate to add color) when embryos were 18-20 hours old and dechorionated the eggs with 0.6% sodium hypochlorite twice (120s and 300s). Inside a sterilized biosafety cabinet maintaining atmospheric sterility, we used a sterile paintbrush to transfer 30-50 sterile eggs into vials prepared with 7.5 mL of diet. For experiments without vitamin supplements, 7.5 mL of Y-G, omitting the acid preservative, was pipetted into 50-mL centrifuge tubes (Trueline, PID0782096), autoclaved, and cooled. For experiments with vitamin supplements, the vitamins were pipetted into sterile 50 mL centrifuge tubes, then mixed with 7.5 mL autoclaved and cooled Y-G diet, omitting the acid preservative. We used vitamins at concentrations reported previously (Wong et al., 2014) . Thiamine (T4625-25G, Sigma-Aldrich; 21 μL or 0.5 mg/mL ddH _2_ O stock, filter sterilized), folic acid (15445239-5G, Thermo Scientific Alfa Aesar; 67.5 μL of 1mg/mL ddH _2_ 0/7 drops NaOH stock, filter sterilized), pantothenic acid (21210-5G-F, Sigma-Aldrich; 16.2 μL of 5 mg/mL ddH _2_ O stock, filter sterilized), and riboflavin (132350250-25G, Thermo Scientific Acros Organics; 10.5 μL of 0.5 mg/mL ddH _2_ O stock, autoclaved). The riboflavin dissolved incompletely and was mixed vigorously immediately before pipetting into tubes.

Separately, individual bacterial strains were streaked for isolation on solid MRS medium (MRS medium, Criterion, C5932), grown in overnight liquid culture in liquid MRS, washed once in phosphate-buffered saline (8 g NaCl, 0.2 g KCl, 1.44 g Na _2_ HPO _4_ , 2.4 g KH _2_ PO _4_ ) and normalized in PBS to OD _600_ = 0.1. Fifty μL of the bacterial suspension was inoculated to sterile eggs within 4 hours of when the eggs were collected. For each experiment at least 4 replicate vials were picked for each condition on at least 3 separate days.

**FlyPAD dietary preference assay.** We measured fly DPY using a flyPAD assay as in our previous work (Call et al., 2022) . We aspirated CO _2_ -anesthetized female flies to empty, sterile vials for 3 h to synchronize their appetite. Then each fly was transferred individually to a separate flyPAD arena (Itskov et al., 2014) . Each flyPAD arena contained two diet troughs that detect each time a fly eats from either trough as a change in capacitance of the trough (measured as a ‘sip’). We prepared the arena by placing 6 μL of Y-G diet into the left well, and 6 μL of yeast-restricted (5% yeast, 10% glucose, 2% agar) diet into the right well. In both cases, the acid preservative was omitted from the diet. FlyPAD modules with flies were placed into a Percival upright incubator at 25°C and 75% humidity and feeding was recorded for 60 minutes. Separately, we homogenized and dilution plated 5 vial-matched male siblings from each vial on mMRS to check for microbiota contamination. If the siblings displayed greater than 100 CFU fly ^-1^ of a contaminating microbe, then data recorded from flies in that vial were discarded. Data were also discarded if both wells registered 0 sips or if at least 1 well registered >1,800 sips. Treatment-specific differences in fly DPY were determined using a generalized linear mixed effects model with a binomial family in R (Bates et al., 2015) . The response variable was the sip count from each fly on Y-G and trial diet. The unique combination of bacterial strain and thiamine supplementation, or the identity of the supplemented B vitamin, was the main effect. Experimental replicate, defined as the batch that measured fly DPY in 48 distinct flyPAD arenas, was included as a random effect. Significant differences between conditions were determined by a post hoc Tukey test and are presented as compact letter displays (Hothorn et al., 2008) . The figure panels report the mean and standard error of the mean of the preference index, which is a value extracted from the model as a linear prediction for each inputted value.

## Reagents

**Table:** 

**Reagent Type**	**Name**	**Additional information**	**Source**
Fly Strain	*Drosophila melanogaster*	CantonS	Mariana Wolfner
Bacterial Strain	*Acetobacter fabarum*	Afa2	White et al., 2018
Bacterial Strain	*iscS*	Plate95-b2	White et al., 2018
Bacterial Strain	*oprB*	Plate6-b9	White et al., 2018
Bacterial Strain	permease mutant	Plate60-a1	White et al., 2018
Bacterial Strain	*thiE*	Plate96-g11	White et al., 2018
